# Socioeconomic inequalities in under-five mortality in rural Bangladesh: evidence from seven national surveys spreading over 20 years

**DOI:** 10.1186/s12939-017-0693-9

**Published:** 2017-11-13

**Authors:** Asiful Haidar Chowdhury, Syed Manzoor Ahmed Hanifi, Mohammad Nahid Mia, Abbas Bhuiya

**Affiliations:** 1 Health Systems and Population Studies Division, Level 7, icddr,b, 68 Shaheed Tajuddin Ahmed Sarani, Mohakhali, Dhaka, 1212 Bangladesh; 2 Partners in Population and Development, Plot 17/B&C, Block F, Sher-E-Bangla Nagar, Administrative Zone, Agargaon, Dhaka, 1207 Bangladesh

**Keywords:** Bangladesh, Socioeconomic inequalities, Under-five mortality, Rural area, BDHS

## Abstract

**Background:**

Socioeconomic inequality in health and mortality remains a disturbing reality across nations including Bangladesh. Inequality drew renewed attention globally. Bangladesh though made impressive progress in health, it makes an interesting case for learning. This paper examined the trends and changing pattern of socioeconomic inequalities in under-five mortality in rural Bangladesh. It also examined whether mother’s education had any effect in reducing socioeconomic inequalities.

**Methods:**

Data from rural samples of seven Bangladesh Demographic Health Surveys, carried out so far, were used. Children born alive during 5 years preceding the surveys were included in the analysis. Univariate, bivariate and multivariate analyses were carried out.

**Results:**

Under-five mortality rate steadily declined over the years from 128/1000 in 1994 to 48 in 2014. Females had 8% lower mortality rates than males. Children of mothers with no schooling had 1.88 times higher mortality than those whose mother had six or more years of schooling. Similarly, children from low asset category households had on an average 1.17 times higher mortality rate than those from high asset category households. Inequality by mother’s education disappeared in the recent years, and inequality by household socioeconomic condition persisted all through. The pattern of inequality by sex, mother’s education, and household socioeconomic status was not changed statistically significantly over the years, and mothers’ education did not reduce socioeconomic inequalities.

**Discussion:**

The reduction in mortality was consistent with changes in the proximate determinants of child survival in the country. Proximate determinants included maternal factors, environmental contamination, nutrient deficiency, personal illness control, and injury. Health and population programmes have been effective in increasing immunization coverage, use of ORS for managing diarrhoeal diseases, and increasing contraceptive use. Development activities on the other hand raised the literacy, especially among females, demand for modern health services, and reduction of poverty. However, socioeconomic inequality still exists in both under-five mortality and proximate determinants of child survival.

**Conclusions:**

The socioeconomic inequality in under-five mortality is showing resistance against further reduction. An assessment of the adequacy of the existing programmes taking the proximate determinants of child survival into consideration will be useful for further improvement.

## Background

Socioeconomic disparity in health and mortality has been a disturbing reality for our societies irrespective of level of development [1–3] and Bangladesh has not been any exception [3–7]. Though health services including child health care by the public sector are free-of-cost in many settings poor access them in lower proportion compared to better offs for they are also less educated and are known to have cultural and social barriers to access health services [8]. In recent times the inequality in health issues drew renewed attention globally with its explicit mention as development goals in global agenda, such as Millennium Development goals (MDGs) and Sustainable Development Goals (SDGs) [9]. The Commission on Social Determinants of Health has been successful in generating discussion around the topic and in influencing global policy and strategic agenda. What remained to be unclear is how best the disparities can be reduced to an acceptable level. Developing nations, like Bangladesh, while made significant progress in improving health and lowering level of mortality, inequalities in health and mortality persists [4, 10, 11]. Though Bangladesh has made progress towards reducing urban-rural and regional inequalities in under-five mortality but socioeconomic inequalities continue to persist [12–14]. Studies carried out in Matlab, Bangladesh, with prospective health and demographic data, revealed that usual health intervention programmes do not reduce poor-rich gap [15] but poverty alleviation programme does [16]. Bangladesh experience has been important where many poverty alleviation programmes, especially microfinance, targeted to the most disadvantaged have been implemented with a view to improve socioeconomic condition of the disadvantaged and reduce socioeconomic inequalities. Impact of such development programmes in reducing socioeconomic inequalities has been examined and found to have health inequality reducing effects [16, 17]. Alongside the microfinance programmes, Bangladesh also pursued free universal primary education programme with emphasis on girls backed by cash/material incentives for education resulting in sharp rise in female education [18]. Also notable was the expansion of free immunization programme, family planning services, and primary healthcare by the public sector to take services to doorsteps of people, especially in rural areas resulting in near universal immunization coverage, and increased use of contraceptives [19].

Mothers education, one the many non-health factors that determined child survival, has been found to be very important across nations even in settings, including Bangladesh, with limited health services and socioeconomic development [4, 7, 11, 20–24]. It was argued that education of mothers even of low level empowers the mothers to be effective in influencing decisions for healthcare, ensuring better health and survival of children [21, 25]. It is postulated that maternal education also inculcates modern health knowledge, beliefs and practices, improves the effectiveness of health behaviour such as feeding practices and other child care; and changes the mother’s role within the family, enabling her to take the necessary measures to promote child health, including effective use of modern health services [20].

Maternal education, especially secular education, is not only the single most important factor for improved child survival, it is also a mainstream element of life in contemporary societies, unlike microfinance for example. Thus, it is important to know and understand whether a maternal education, a public goods, reduces socioeconomic inequalities in health and survival of children. Among the socioeconomic factors, as mentioned above, sex of children and household socioeconomic status have been found to be important [6, 12, 13, 16, 17].

Given the socioeconomic inequality in under-five mortality and importance of mother’s education, we have examined the trend of socioeconomic inequality in under-five mortality in rural Bangladesh using data from seven national surveys spreading over 20 years. We also examined whether mother’s education had an effect in reducing socioeconomic inequalities.

## Methods

### Data and variables

Data for this paper came from the seven Bangladesh Demographic Health Surveys (BDHS) carried out so far. The first BDHS was carried out during 1993–94 and the most recent one was in 2014. BDHSs have been based on nationally representative samples of women of age 15–49 years. Details of the methods along with other necessary information can be found in the BDHS reports [6].

Children born alive to rural women during 5 years preceding the date of interview from all the surveys were included in this analysis. Each child formed a data record with information on year of survey, sex of the child, household asset score, mother’s education, age of child on the day of interview who survived till to interview, age at death for dead children, and survival status of the child at the time of data collection.

Asset score, a commonly used indicator of household socioeconomic status, was categorized into three (low, middle, and high) with approximately one third of the households in each category. Mother’s education was categorized into three: no education, primary enrolled, and secondary enrolled. Primary and secondary schooling are the two lowest levels in Bangladesh secular education system. Primary level included 1–5 years of schooling, and secondary included 6–10 years. All the variables were treated as categorical variables in multivariate analysis. Reference categories for multivariate analysis are indicated in the relevant tables.

### Analysis

Under-five mortality rate was calculated by using life table approach, which used mortality probabilities for small age segments, to be consistent with the BDHSs’ published rates. Detail of this approach used in DHS data analysis has been reported elsewhere [26]. Trends in under-five mortality and its socioeconomic inequality were examined by using the mortality rates and their rate-ratios [27]. Wilcoxon Gehan test was carried out to determine whether under-five mortality rate differ significantly between category of independent variables.

Examination of statistical significance of the association of an independent variable with under five mortality controlling the effect of other independent variables was examined by Cox’s Proportional Hazard Regression analysis [28]. Cox’s Proportional Hazard Regression analysis was also used to examine whether the socioeconomic inequalities have changed over time by including products of year and various socioeconomic variables (i.e. mother’s education, asset score, sex of child) in other words interaction terms, along with the main effects, including one interaction term at a time. In a similar way, modifying effects of mother’s education in reducing sex and socioeconomic inequalities was assessed by including a product/interaction terms of mother’s education and sex or household socioeconomic status. Inequalities associated with a socioeconomic indicator is considered to have changed/modified if the corresponding regression co-efficient of the interaction term was statistically significant based on Wald chi-square statistics. The Interaction terms to be tested were based on questions to be answered as mentioned earlier, so they were decided a priori. An examination of the degree of association among the independent variables was made by cross-tabulating the independent variables with each other.

Analyses were carried out using SPSS version 20.

## Results

### Univariate analysis

Table 1 presents under-five mortality rates by various social determinants and year of surveys in rural Bangladesh. The overall under-five mortality rate during the period, approximately 1994–2014, was 84.2 per 1000 live birth with highest (128.0) in 1994 and lowest (48.3) in 2014. There was a steady and statistically significant decline in under-five mortality rate over the years with an average decline of 0.013 or 13 per 1000 children in between surveys.

**Table 1:** Under-five mortality rates per 1000 live births and mortality rate ratios by year of surveys and various social determinants

Social Determinants	Year							All
	1994	1997	2000	2004	2007	2011	2014	
Sex								
Male	131.9	120.8	89.8	80.7	74.3	59.6	48.5	87.5
Female	123.9	113.5	81.6	76.7	70.7	44.6	48.1	80.7
Rate Ratio (Female/ Male)	0.939	0.940	0.909	0.950	0.952	0.748	0.992	0.922
* P* value*	0.138	0.444	0.393	0.609	0.404	0.011	0.986	0.009
Mother’s education in years of schooling								
None	143.2	132.9	99.3	99.6	83.6	57.1	61.4	109.5
1–5	106.3	102.8	70.8	67.0	74.7	56.3	47.8	74.8
6+	102.9	73.0	73.9	62.0	62.7	47.1	44.5	58.2
Rate Ratio (None/6+)	1.392	1.821	1.344	1.607	1.333	1.212	1.380	1.881
*P* value*	0.002	0.007	0.042	0.000	0.006	0.221	0.107	0.000
Asset Score								
Low	146.7	105.6	98.0	90.8	72.4	57.7	56.4	84.4
Medium	134.0	125.9	98.1	82.6	71.4	54.4	54.5	96.0
High	110.1	105.1	61.7	63.0	75.8	43.8	32.8	72.1
Rate Ratio (Low/High)	1.332	1.005	1.588	1.441	0.955	1.317	1.720	1.171
*P* value*	0.002	0.047	0.016	0.034	0.559	0.069	0.001	0.000
All	128.0	117.1	85.8	78.8	72.5	52.3	48.3	84.2
*P* value*	0.000							

* Based on Wilcoxon Gehan statistic

As can be seen in Table 1, combining all the years, females had statistically significantly 8% lower mortality rates than males. Speaking of yearly sex differentials, excepting 2011, male and female mortality rates were statistically equal in all the other years.

Children of mothers without any schooling had 88% higher mortality rate than children of mothers with six or more years of schooling when mortality rates of all the years were combined. The statistically significant higher mortality rates among children of mothers with no schooling compared to children of mothers with six or more years of schooling was observed in all the years excepting the latest two surveys. Declining trends of mortality rates were observed among children of mothers in all the three educational categories, the decline though was largest for children of mothers without any schooling.

Children coming from households belonging to the low asset score category had 17% higher mortality rate compared to the one coming from high asset score category households when all the surveys were combined. Mortality rates of children coming from the low asset category households were always statistically significantly higher than those coming from the high asset category households excepting 2007. Over the years the mortality rates had declined for children in all the three categories of households.

### Multivariate analysis

Results of Cox’s Regression analysis are presented in Table 2. It can be seen that year, sex, mother’s education, and household socioeconomic condition (i.e. asset score) had statistically significant association with under-five mortality. The patterns of relationship observed in multivariate analysis were almost similar to results observed in univariate and bivariate analysis. In relative sense the mortality rate in 1994 was 2.3 times of 2014. Male children had a death rate of 1.1 times than female. Children of mothers without any schooling had 1.35 times higher mortality rates than those with mothers having more than 5 years of schooling. Children from low socioeconomic category households had 1.21 time higher mortality than those from high category households.

**Table 2:** Cox’s regression coefficients and hazard ratios (HR) with 95% confidence intervals (CIs) of under-five mortality associated with independent variables in rural Bangladesh, 1994–2014

Independent variables	Coefficients (β_s_)	*P* value	Hazard Ratio (95% CIs)
Year***		0.000	
1994	0.822	0.000	2.274 (1.937–2.669)
1997	0.736	0.000	2.087 (1.765–2.467)
2000	0.486	0.000	1.626 (1.374–1.924)
2004	0.451	0.000	1.570 (1.324–1.862)
2007	0.354	0.000	1.424 (1.191–1.703)
2011	0.084	0.341	1.088 (0.914–1.295)
2014	Reference		1.0
Sex*		0.016	
Male	0.094	0.016	1.098 (1.018–1.185)
Female	Reference		1.0
Mother’s education***		0.000	
None	0.297	0.000	1.345 (1.196–1.514)
1–5 years	0.106	0.076	1.112 (0.989–1.249)
6 years+	Reference		1.0
Asset score**		0.001	
Low	0.193	0.000	1.213 (1.088–1.351)
Middle	0.175	0.001	1.191 (1.078–1.316)
High	Reference		1.0

Note: Significance level *** - *P* < .001; ** - *P* < .01; * - *P* < .05;

The pattern and extent of inequalities by various independent variables over the years did not change statistically. This was confirmed by the lack of significance of the regression co-efficients associated with the interaction terms of year and sex, year and mother’s education, and year and household socioeconomic status (results not presented).

An examination of the effects of mother’s education in reducing sex and socioeconomic inequalities in under-five mortality was made by including interaction term of mother’s education and sex, and mother’s education and socioeconomic status of households, one at a time with the main effect models. The co-efficients associated with both the interaction terms were statistically insignificant (results not presented). This implied that the sex differentials and socioeconomic differentials observed over the period was maintained for children of mothers belonging to all categories of mother’s education. In other words, mother’s education did not change the inequalities in a statistically significant way.

## Discussion

The analysis revealed that the level of under-five mortality in rural Bangladesh has reduced significantly during the last two decades. The negative relationship between household socioeconomic status and mother’s education with childhood mortality has been consistent with findings reported earlier in Bangladesh and other similar settings [4, 7, 23, 27, 29, 30]. Higher male mortality than female is somewhat different than reported earlier, where female children aged 1–4 years had higher mortality than male, and male had higher mortality than female during neonatal period [6, 12, 31]. The present analysis examined under-five mortality in which the neonatal deaths formed the major part due to rapid decline in childhood mortality during age 1–4 years of life. Thus, the higher under five mortality for male than female was due to lower female mortality during neonatal period [6].

The status in socioeconomic inequality in terms of sex, household socioeconomic status, and mother’s education persisted all through during the last two decades, even if the overall under-five mortality has declined steadily. Education of mothers had no significant effect in reducing sex or household socioeconomic status based differentials in under-five mortality over the time, which is somewhat different than findings from an earlier study where mother’s education was found to be more effective for boys than girls [32]. Though population living below poverty line in rural Bangladesh had decreased substantially from 52.3% in 2000 to 35.2% in 2010 [33], nevertheless the socioeconomic inequality in under-five mortality persisted.

Explanation of persistence of inequalities in under-five mortality can be aided by using the proximate determinants framework of child survival [34]. The framework assumed that child survival works through a set of five proximate determinants, which in turn are influenced by socioeconomic factors. The set of proximate determinants included maternal factors, environmental contamination, nutrient deficiency, personal illness control and injury. Thus, it may be of interest to discuss the trends of the proximate determinants along with and socioeconomic factors’ influence on them in case of under-five mortality.

Mother’s age at birth, contraceptive use, birth interval, and fertility rate are a reasonable set of indicators of maternal factors. Total fertility rate in Bangladesh has reduced from 6 in the 70s to 2 in 2014 [6, 11, 35]. Age of mother at first and second births in rural Bangladesh has increased during the last decades. In 1994, the average age at first birth was 17.7 years compared to 18.4 years in 2014. The decline was greater for the mothers of higher socioeconomic class and among those who had five or more years of schooling [6, 13]. Thus, overall decline in under-five mortality might have a component of positive effect of age at birth and child survival, and the benefit was greater for children coming from households belonging to higher socioeconomic groups.

Contraceptive use has increased steadily during the last couple of decades from 40% in 1990 to 62% in 2014 [6, 13, 36]. This has reduced the risk of child mortality associated with higher order births, short birth intervals and large family size, which might have contributed in reducing overall child mortality. The benefit of smaller number of children in terms of child survival is likely to be derived mostly by the children born to educated women and coming from higher socioeconomic households, because educated women and those coming from better off households used family planning methods more than their counterparts from lower level of education and from low socioeconomic households [11] .

Water and sanitation are two important indicators of environmental contamination. There has been a big shift in the sources of water at the household level during the last decades from pathologically unsafe water sources to safe underground water [34, 37]. Again better off households had more access to safe water than worse off households [6]. So was the case with use of safe latrines [6]. Prevalence of severe malnutrition, which used to be a major direct and underlying cause of child mortality, has reduced significantly during the last decades with lower prevalence among children from better off households and those of mothers who had schooling [6, 38, 39] .

Immunization and management of childhood illnesses are two important personal illness control measures, which have contributed enormously in reducing under-five mortality. Bangladesh’s success in increasing childhood immunization coverage from near 6% in mid 80s to above 80% now has contributed enormously in reducing under-five mortality [6, 11] . Use of ORS (Oral rehydration solution) for management of diarrhoea, a common childhood illnesses with myriad health consequences, has increased from very low level in early 80s to over 80% now at the household level [6, 11]. This also has contributed in reducing childhood mortality. Unfortunately, all of the personal illness control measures have socioeconomic gradients with higher use rate among children from better off households and of mothers with education, resulting in socioeconomic inequalities in under-five mortality. Among injuries drowning is emerging to be a major cause of deaths among under-five children. Male children had higher drowning death rates than female, and children from lower socioeconomic households had higher mortality from drowning than their counterparts from better off households [40].

Thus, the overall improvement in under-five mortality situation in Bangladesh was an outcome of improvement of the situation in terms of proximate determinants of child survival along with reduction in level of poverty and increased educational level, especially of females. The prevailing socioeconomic inequalities in under-five mortality at the current lower level of mortality was a reflection of the prevalence of socioeconomic inequalities in the proximate determinants favouring the better off in terms of mothers’ education and household socioeconomic status. Absence of sex differentials in mortality is a reflection of achieving equalities in the use of personal illness control measures, such as immunization and treatment of common illnesses and use ORS, for boys and girls [11].

## Conclusions

Bangladesh has made tremendous progress in reducing under-five mortality and inequality to some extent during last two decades but the socioeconomic inequality in under-five mortality is showing resistance against further reduction. An assessment of adequacy of existing programmes taking proximate determinants of child survival into consideration will be useful. Future programmes and policies should have an explicit goal of reducing socioeconomic inequality and performance of such policies and programmes should be monitored for their efficacy in reducing inequalities.

### Strengths and limitations of the study

The major strength is that the paper is based on nationally representative rural sample covering a period of 20 years and the findings are applicable for whole of rural Bangladesh. The present analysis did not adjust for autocorrelation inherent in data of this kind, which is a limitation of the analysis. Lack of understanding of causality is another limitation for the findings are based on degree of association between under-five mortality and socioeconomic determinants and hence do not provide an understanding of causality.

## Abbreviations

AS: Asset Score
BDHS: Bangladesh Demographic Health Survey
CIs: Confidence intervals
HR: Hazard ratio
MDGs: Millennium Development Goals
MEASURE DHS: Monitoring and Evaluation to Assess and Use Results Demographic and Health Surveys
MED: Mother’s education
NIPORT: National Institute of Population Research and Training
ORS: Oral rehydration solution
SDGs: Sustainable Development Goals
U5MR: Under-five mortality rate
